# Understanding Gender-Bias in Critically Ill Patients With COVID-19

**DOI:** 10.3389/fmed.2020.564117

**Published:** 2020-09-25

**Authors:** Mustapha Chamekh, Georges Casimir

**Affiliations:** ^1^Inflammation Unit, Laboratory of Pediatric Research, Université Libre de Bruxelles (ULB), Brussels, Belgium; ^2^Department of Pulmonology, Faculty of Medicine, Queen Fabiola University Children's Hospital (HUDERF), Université Libre de Bruxelles (ULB), Brussels, Belgium

**Keywords:** COVID-19, gender-bias, severe cases, inflammatory markers, pathological inflammation

## Introduction

Understanding gender-bias in COVID-19 infection is of paramount importance, not only from an epidemiological point of view but also in terms of clinical management. It is largely acknowledged that men and women exhibit a different degree of susceptibility to a number of infectious diseases and that this is far from being solely an exposure bias. In acute infections, women generally have a better prognosis compared to men, due to their robust and effective immune defense. Recent clinical and demographical studies on patients with COVID-19 have yielded convergent findings indicating that men might be more severely affected than women. However, in most studies, the data are not stratified by sex, thereby limiting our understanding of how gender impacts outcomes. In this article, we discuss observational clinical studies arguing that the rate of severe cases requiring intensive care is higher in men compared to women. We highlight the importance of using gender as a biological variable to determine whether men and women exhibit distinct clinical and biological characteristics that could explain gender-bias. As the hallmark of the disease severity is excessive inflammation in response to viral infection, we make inferences based on the sexual dimorphism of the inflammatory response seen in different infectious models and we focus our discussion on major inflammatory processes that are relevant to COVID-19.

## Evidence for Gender-Bias in COVID-19: the Lack of Data Stratified by Sex

Recent studies on severe acute respiratory syndrome caused by COVID-19 (SARS-CoV2) indicate that men are more severely affected than women. This applies to patients from different regions of the world including China where the pandemic started, Europe, and the USA (1–10). These clinical studies reported demographical (number of positive cases, age, and sex) and classical clinical parameters including symptoms (fever, headaches, sore throat, rhinorrhea, shortness of breath, diarrhea, and nausea, etc.), comorbidities (hypertension, diabetes, and cardiovascular disease, etc.), chest x-ray and CT findings, duration of hospitalization, number of cases requiring intensive care, and outcomes. Approximately 60–70% of the critically ill patients that required intensive care were men. Some of these studies also reported laboratory findings including the number of peripheral blood immune cell subsets and/or cytokine levels (2, 3, 8–10). However, the data recorded in these reports were not stratified by sex, thus hampering a full interpretation of gender-bias.

Among these reports, a multicenter study by Grassieli et al. (6) on baseline characteristics and the outcomes of 1,591 patients with severe COVID-19 admitted to ICUs in Lombardy, Italy, highlights a remarkable gender-bias in infection rates, with males representing 82% of cases, strengthening previous observations that indicate that being male is a risk factor. We think this rate—the highest ever reported in a hotspot of COVID-19—warrants an in-depth analysis as it covers a large number of patients. This study is informative and includes the basic characteristics of critically ill patients in this hotspot, but it did not address whether men and women exhibit distinct clinical features, thereby limiting understanding of how gender impacts outcomes. To further understand the strong gender-bias indicated by this study, it would have been interesting to stratify the data by gender to determine whether marked differences between men and women emerge. The high rate of poor prognosis observed in men may have been balanced by potential differences in comorbidity between men and women. Among the 26% of mortality reported in this study, the proportion of men is likely to be higher than that of women, but it would be interesting to know whether the mortality rate in men reached a rate as high as 80%. Interestingly, this report reveals that severe cases do not only concern the elderly but are observed in all age groups. We noticed that the ratio of men vs. women under 60 was slightly higher compared to that for patients over 60, suggesting that gender-related physiological factors rather than age, play a cardinal role in disease severity.

## Cross-Analyzing Clinical Scores and Inflammatory Markers According to Sex: a Key to Unraveling Factors Underlying Gender-Bias in COVID-19

Growing evidence suggests that the disease severity in SARS-CoV2 is mainly caused by an overwhelming inflammatory reaction in response to viral infection, likely attributed to a cytokine storm and massive recruitment of mononuclear cells within lung tissues [reviewed in (11–13)]. Studies have reported that patients under intensive care produced higher levels of inflammatory cytokines and chemokines (2, 8, 9), with IL-6 being a valuable candidate for monitoring severe cases (10). Yet, no indication was provided about the distribution of these inflammatory cytokines according to gender. The excessive release of pro-inflammatory cytokines and chemokines by immune and probably non-immune cells like pulmonary epithelial cells could induce severe lung injury leading to acute respiratory distress syndrome-ARDS-mediated death (14). This is consistent with earlier investigations on related viral infections (SARS-CoV1 and MERS-CoV) showing increased amounts of circulating pro-inflammatory cytokines and extensive lung damage (15, 16). It would be interesting to analyze a wide range of inflammatory markers in male and female patients with COVID-19 to identify the role of the sexually dimorphic inflammatory response in gender-bias. A recent preliminary study outlined gender-bias in morbidity and mortality in China and highlighted in a limited number of cases differences in few biological parameters including neutrophils (17). On the other hand, studies reported that lymphopenia and the infiltration of mononuclear cells in different organs were associated with disease severity (18). Whether men and women exhibit contrasting scores remains to be demonstrated.

## Discussion

It is largely agreed that the immune inflammatory response is a double-edged sword. Although it helps fight the invasion of the pathogen, it may ultimately come at a cost, causing damage to the host tissue when it is excessive and not tightly regulated. Differences in the inflammatory response type and magnitude according to gender have been observed in infectious and non-infectious inflammatory diseases across all ages including pre-pubescent children (19, 20), and the number of X chromosomes was demonstrated to be key in this respect (21, 22). This stresses the need to include gender as a biological variable for optimal analysis of data recorded from COVID-19 patients.

Multiple factors can impact gender-bias in infection-induced inflammatory diseases. Although the exposure bias associated with gender-dependent behavior cannot be ruled out, accumulating evidence suggests the importance of physiological factors such as sex hormones and sex-specific genetic architecture (23). Sexual hormones were demonstrated to modulate the immune inflammatory response and thereby the course of infection in certain pathological models (24). However, it should be noted that in many infectious diseases, sexual dimorphism was observed across all ages including non-pubescent children, suggesting that X chromosome-linked genetic architecture plays a prominent role. Indeed, females are endowed with a genetic fitness offering crucial advantages in immune defense mechanisms, as they display mosaic cells expressing two X-linked gene alleles, allowing them to better cope with diseases associated with mutations naturally occurring on the X chromosome (25). Moreover, the gene inactivation process that occurs on one of the two X chromosomes early in embryogenesis is thought to be variable and not complete, which may lead to the differential expression of the number of X chromosome-linked genes between males and females (26, 27). This can be critical regarding the magnitude of the inflammatory response in SARS-CoV2 since the X chromosome encompasses several immune genes necessary for the development and homeostasis of the inflammatory response (28, 29). One can cite as examples the pathogen recognition receptors-PRRs (Toll-Like receptors TLR7 and TLR8) that sense single strand RNA from the virus and induce a protective type I interferon response, and the IL-1 receptor–associated kinase 1 (IRAK-1), a key component of NF-kB inflammatory signaling pathways. Overexpression and hyper-activation of NF-kB may explain the high amount of pro-inflammatory cytokines seen in COVID-19 infection, with PRRs-MyD88 axis probably playing a major role. Of note, is the fact that in chronic inflammatory models where females are more susceptible compared to men, studies have demonstrated that TLR7 and IRAK-1 genes can escape the X chromosome inactivation process resulting in their strong activation in females (30, 31). However, in infection-induced acute inflammatory diseases like COVID-19 where females have a better prognosis, this can be regarded as an immune advantage enabling women to defend themselves better against acute infection.

**Figure 1:**
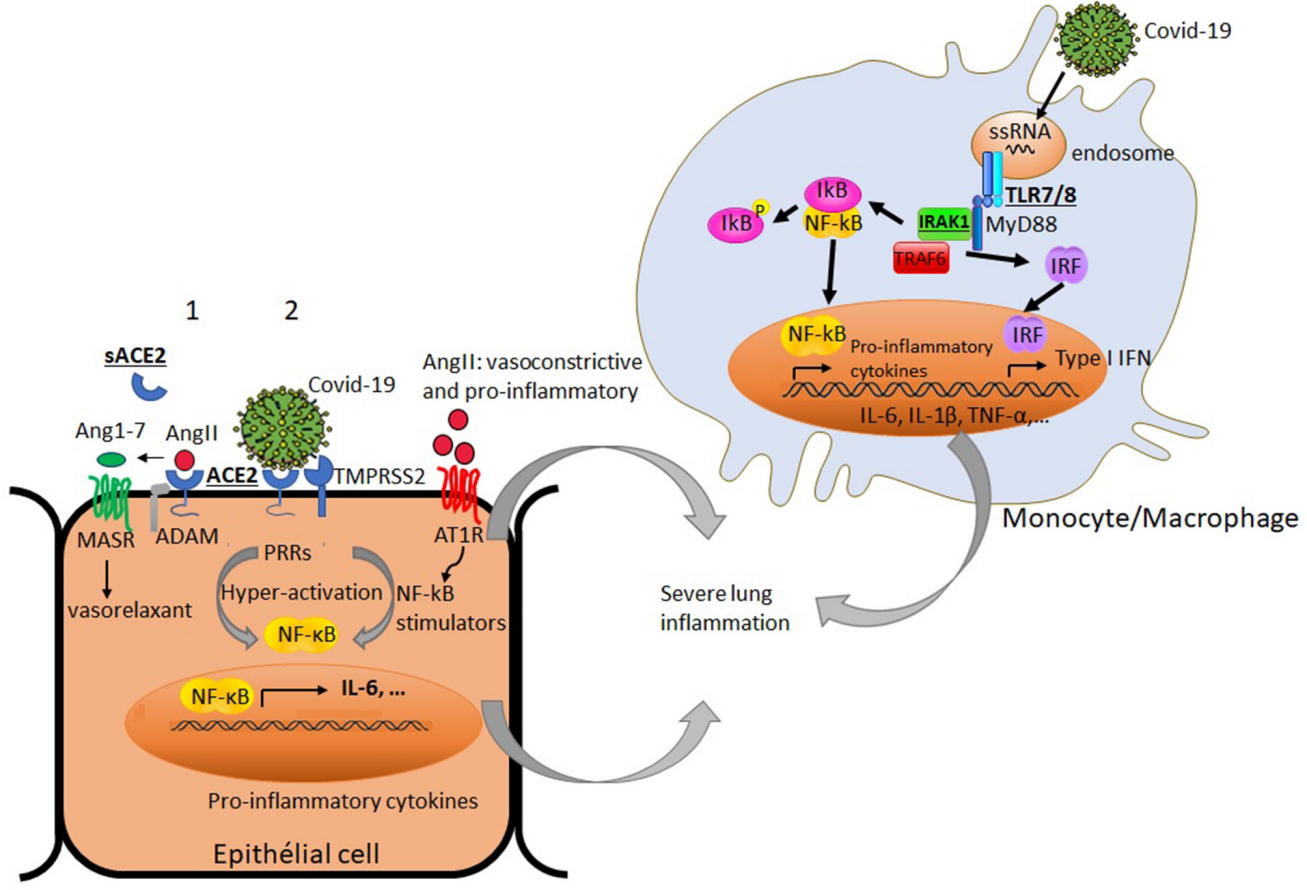
Simplified diagram of potential factors in immune and non-immune cells that may differently impact productive COVID-19 infection and virus-induced airway inflammation in males and females: Under basal condition (1), angiotensin converting enzyme 2 (ACE2) binds angiotensin II (AngII), and prevents its adverse vasoconstrictor and profibrotic effects by its conversion to angiotensin 1-7 (Ang1-7). The later triggers signaling through G-protein coupled Mas receptor (MASR) allowing vasodilatation. The proteolytic shedding of ACE2 by a disintegrin and metalloproteinase (ADAM) can lead to a catalytically active soluble form of ACE2 (sACE2). Under viral infection (2), SARS-Cov2 uses ACE2 as a cell entry receptor and the transmembrane serine protease 2 (TMPRSS2) to allow the fusion of cell and viral membranes for productive infection. The endocytosis of ACE2 together with SARS-Cov2 results in the reduction of ACE2 on cells and an increase of serum AngII. AngII acts not only as a vasoconstrictor but also as a pro-inflammatory mediator through binding to angiotensin II receptor type 1 (AT1R) and the processing of NF-kB stimulators (soluble TNF-α and IL-6 receptors) and IL-6 amplifier. The activation of NF-kB and the production of inflammatory cytokines are enhanced by SARS-CoV-2 itself via pattern recognition receptors (PPRs). In innate immune cells like monocytes, the interaction of the endosomal TLR7/8 with single stranded RNA from the virus can lead to the recruitment of adaptor molecules like MyD88 and the activation of signaling molecules including IL-1 receptor associated kinase-1 (IRAK-1). This culminates with the activation and translocation of NF-kB to the nucleus and subsequent production of the inflammatory mediators (TNF-α, IL-1β, IL-6…) and types I IFN gene products, thereby amplifying the lung inflammation. Some X-chromosome-linked factors (ACE2, IRAK1, TLR7/8) that could differently influence productive COVID-19 infection and the magnitude of the inflammatory response in males and females are underlined.

COVID-19 uses the angiotensin converting enzyme II (ACE2) expressed in different organ tissues as a cell entry receptor (32, 33). Interestingly, ACE2 was shown to have a crucial role in preventing severe acute lung injury (34, 35). It is believed that cell surface expression of ACE2 in patients with COVID-19 is downregulated following its endocytosis with the virus, hence reducing its accessibility to its natural ligand angiotensin II (AngII). Consequently, this may lead to the accumulation of circulating AngII, a vasoconstrictor endowed with a pro-inflammatory potential (11, 36). As the ACE2 gene is located on the X chromosome, its potential escape from the X chromosome inactivation process could favor its differential expression in males and females. Whether ACE2 expression varies according to gender remains unclear (37, 38). An in-depth analysis of ACE2 expression within different tissues was reported and found to be up-regulated by type I interferons (39). It has been also shown that the expression of ACE2 transcript in the human nasal epithelium is age-dependent but sex-independent (40). Further investigations on ACE2 expression at transcript and protein levels would be of interest to decipher its potential implication in the gender-bias of COVID-19-induced inflammatory response. It is therefore worth noting that sex-based differences in the regulation of the renin-angiotensin pathway and the resulting activation of NF-kB signaling, which is amplified in the course of COVID-19 infection, could lead to differences in the exacerbation of the lung inflammation between males and females (Figure 1).

On the other hand, there is evidence that a soluble form of ACE2 which is cleaved from the membrane by peptidases, called sheddases (ADAM 10 and ADAM 17), is catalytically active (41, 42). It has been shown that individuals with diabetes, renal diseases, or heart failure have altered serum levels of ACE2 (43, 44). Moreover, when gender bias was taken into account, plasma ACE2 concentrations were found to be higher in men than in women (45). Given the fact that such diseases are comorbidities associated with severe outcomes in COVID-19, this leads to speculation that serum levels of ACE2 may influence productive infection of SARS-CoV-2. Although studies reported the shedding of ACE2 *in vivo*, its physiological importance in COVID-19 infection remains unclear. It is tempting to consider that high serum ACE2 levels may reflect a higher level of its membrane bound counterpart in pulmonary tissues. In this case, increased amounts of ACE2 in males could explain their higher susceptibility to severe SARs-CoV-2. On the other hand, Li et al. (46) showed *in vitro* that a soluble form of ACE2 can block the binding of the SARS-CoV spike protein to its receptor. To what extent the blocking of binding of the virus to its membrane receptor ACE2 by a soluble form of ACE2 occurs *in vivo* is yet to be unraveled. This suggests that the balance between soluble and membrane-bound ACE2 in males and females with comorbidities associated with severe COVID-19 severity might influence the viral pathogenesis.

In conclusion, the factors and mechanisms underlying sex and gender-bias in critically ill patients with COVID-19 remain scarce. The use of sex as a biological variable to depict the distribution of clinical and biological data will fill gaps in our understanding of why men are more severely affected than women. This may help to optimize clinical monitoring and strengthen gender medicine practice.

## Author Contributions

MC reviewed the litterature and drafted the manuscript. GC reviewed the manuscript. All authors contributed to the article and approved the submitted version.

## Conflict of Interest

The authors declare that the research was conducted in the absence of any commercial or financial relationships that could be construed as a potential conflict of interest.

## References

[1] Huang C Wang Y Li X Ren L Zhao J Hu Y. Clinical features of patients infected with 2019 novel coronavirus in Wuhan, China. Lancet. (2020) 395:497–506. 10.1016/S0140-6736(20)30183-5 31986264 PMC7159299

[2] Chen N Zhou M Dong X Qu J Gong F Han Y. Epidemiological and clinical characteristics of 99 cases of 2019 novel coronavirus pneumonia in Wuhan, China: a descriptive study. Lancet. (2020) 395:507–13. 10.1016/S0140-6736(20)30211-7 32007143 PMC7135076

[3] Wang D Hu B Hu C Zhu F Liu X Zhang J. Clinical characteristics of 138 hospitalized patients with 2019. novel coronavirus-infected pneumonia in Wuhan, China. JAMA. (2020) 323:1061–9. 10.1001/jama.2020.1585 32031570 PMC7042881

[4] Yang X Yu Y Xu J Shu H Xia J Liu H. Clinical course and outcomes of critically ill patients with SARS-CoV-2 pneumonia in Wuhan, China: a single-centered, retrospective, observational study. Lancet Respir Med. (2020) 8:475–81. 10.1016/S2213-2600(20)30079-5 32105632 PMC7102538

[5] Spiteri G Fielding J Diercke M Campese C Enouf V Gaymard A. First cases of coronavirus disease 2019. (COVID-19) in the WHO European region, 24 january to 21 february 2020. Euro Surveill. (2020) 25:2000178. 10.2807/1560-7917.ES.2020.25.9.2000178 32156327 PMC7068164

[6] Grasselli G Zangrillo A Zanella A Antonelli M Cabrini L Castelli A. Baseline characteristics and outcomes of 1591. patients infected with SARS-CoV-2 admitted to ICUs of the Lombardy Region, Italy. JAMA. (2020) 323:1574–81. 10.1001/jama.2020.5394 32250385 PMC7136855

[7] Richardson S Hirsch JS Narasimhan M Crawford JM McGinn T Davidson KW. Presenting characteristics, comorbidities, and outcomes among 5700 patients hospitalized with COVID-19 in the New York City area. JAMA. (2020) 323:2052–9. 10.1001/jama.2020.6775 32320003 PMC7177629

[8] Chen G Wu D Guo W Cao Y Huang D Wang H. Clinical and immunological features of severe and moderate coronavirus disease 2019. J Clin Invest. (2020) 130:2620–9. 10.1172/JCI137244 32217835 PMC7190990

[9] Qin C Zhou L Hu Z Zhang S Yang S Tao Y. Dysregulation of immune response in patients with COVID-19 in Wuhan, China. Clin Infect Dis. (2020) 71:762–8. 10.1093/cid/ciaa248 32161940 PMC7108125

[10] Liu T Zhang J Yang Y Ma H Li Z Zhang J. The role of interleukin-6 in monitoring severe case of coronavirus disease (2019). EMBO Mol Med. (2020) 12:e12421. 10.15252/emmm.202012421 32428990 PMC7280589

[11] Hirano T Murakami M. COVID-19: a new virus, but a familiar receptor and cytokine release syndrome. Immunity. (2020) 52:731–3. 10.1016/j.immuni.2020.04.003 32325025 PMC7175868

[12] Tay MZ Poh CM Rénia L MacAry PA Ng LFP. The trinity of COVID-19: immunity, inflammation and intervention. Nat Rev Immunol. (2020) 20:363–74. 10.1038/s41577-020-0311-8 32346093 PMC7187672

[13] Merad M Martin JC Pathological inflammation in patients with COVID-19: a key role for monocytes and macrophages. Nat Rev Immunol. (2020) 20:355–62. 10.1038/s41577-020-0331-4 32376901 PMC7201395

[14] Mehta P McAuley DF Brown M Sanchez E Tattersall R Manson JJ. COVID-19: consider cytokine storm syndromes and immunosuppression. Lancet. (2020) 395:1033–4. 10.1016/S0140-6736(20)30628-0 32192578 PMC7270045

[15] Wong CK Lam CW Wu AK Ip WK Lee NL Chan IH. Plasma inflammatory cytokines and chemokines in severe acute respiratory syndrome. Clin Exp Immunol. (2004) 136:95–103. 10.1111/j.1365-2249.2004.02415.x 15030519 PMC1808997

[16] Mahallawi WH Khabour OF Zhang Q Makhdoum HM Suliman BA. MERS-CoV infection in humans is associated with a pro-inflammatory Th1 and Th17 cytokine profile. Cytokine. (2018) 104:8–13. 10.1016/j.cyto.2018.01.025 29414327 PMC7129230

[17] Jin JM Bai P He W Wu F Liu XF Han DM. Gender differences in patients with COVID-19: focus on severity and mortality. Front Public Health. (2020)8:152. 10.1101/2020.02.23.20026864 32411652 PMC7201103

[18] Xu Z Shi L Wang Y Zhang J Huang L Zhang C. Pathological findings of COVID-19 associated with acute respiratory distress syndrome. Lancet Respir Med. (2020) 8:420–2. 10.1016/S2213-2600(20)30076-X 32085846 PMC7164771

[19] Casimir GJ Lefèvre N Corazza F Duchateau J. Sex and inflammation in respiratory diseases : a clinical viewpoint. Biol Sex Differ. (2013) 4:1–9. 10.1186/2042-6410-4-16 24128344 PMC3765878

[20] Chamekh M Casimir G. Editorial: sexual dimorphism of the immune inflammatory response in infectious and non-infectious diseases. Front Immunol. (2019) 10:107. 10.3389/fimmu.2019.00107 30804936 PMC6371857

[21] Lefèvre N Corazza F Valsamis J Delbaere A De Maertelaer V Duchateau J. The number of X chromosomes influences inflammatory cytokine production following Toll-like receptor stimulation. Front Immunol. (2019) 10:1052. 10.3389/fimmu.2019.01052 31143188 PMC6521177

[22] Laffont S Rouquié N Azar P Seillet C Plumas J Aspord C. X-Chromosome complement and estrogen receptor signaling independently contribute to the enhanced TLR7-mediated IFN-α production of plasmacytoid dendritic cells from women. J Immunol. (2014) 193:5444–52. 10.4049/jimmunol.1303400 25339659

[23] Chamekh M Deny M Romano Lefèvre N Corazza F Duchateau J. Differential susceptibility to infectious respiratory diseases between males and females linked to sex-specific innate immune inflammatory response. Front Immunol. (2017) 8:1806. 10.3389/fimmu.2017.01806 29321783 PMC5733536

[24] Vom Steeg LG Klein SL. Sex and sex steroids impact influenza pathogenesis across the life course. Semin Immunopathol. (2019) 41:189–94. 10.1007/s00281-018-0718-5 30298431 PMC6370518

[25] Lessing D Anguera MC Lee JT. X chromosome inactivation and epigenetic responses to cellular reprogramming. Annu Rev Genomics Hum Genet. (2013) 14:85–110. 10.1146/annurev-genom-091212-153530 23662665

[26] Carrel L Willard HF. X-inactivation profile reveals extensive variability in X-linked gene expression in females. Nature. (2005) 434:400–4. 10.1038/nature03479 15772666

[27] Tukiainen T Villani AC Yen A Rivas MA Marshall JL Satija R. Landscape of X chromosome inactivation across human tissues. Nature. (2017) 550:244–8. 10.1038/nature24265 29022598 PMC5685192

[28] Fish EN. The X-files in immunity: sex-based differences predispose immune responses. Nat Rev Immunol. (2008) 8:737–44. 10.1038/nri2394 18728636 PMC7097214

[29] Libert C Dejager L Pinheiro I. The X chromosome in immune functions: when a chromosome makes the difference. Nat Rev Immunol. (2010) 10:594–604. 10.1038/nri2815 20651746

[30] Spolarics Z Peña G Qin Y Donnelly RJ Livingston DH. Inherent X-linked genetic variability and cellular mosaicism unique to females contribute to sex-related differences in the innate immune response. Front Immunol. (2017) 8:1455. 10.3389/fimmu.2017.01455 29180997 PMC5694032

[31] Souyris M Cenac C Azar P Daviaud D Canivet A Grunenwald S. TLR7 escapes X chromosome inactivation in immune cells. Sci Immunol. (2018) 3:eaap8855. 10.1126/sciimmunol.aap8855 29374079

[32] Zhou P Yang XL Wang XG Hu B Zhang L Zhang W. A pneumonia outbreak associated with a new coronavirus of probable bat origin. Nature. (2020) 579:270–3. 10.1038/s41586-020-2012-7 32015507 PMC7095418

[33] Hoffmann M Kleine-Weber H Schroeder S Krüger N Herrler T Erichsen S. SARS-CoV-2 cell entry depends on ACE2 and TMPRSS2 and is blocked by a clinically proven protease inhibitor. Cell. (2020) 181:271–80.e8. 10.1016/j.cell.2020.02.052 32142651 PMC7102627

[34] Imai Y Kuba K Rao S Huan Y Guo F Guan B. Angiotensin-converting enzyme 2 protects from severe acute lung failure. Nature. (2005) 436:112–6. 10.1038/nature03712 16001071 PMC7094998

[35] Kuba K Imai Y Rao S Gao H Guo F Guan B. A crucial role of angiotensin converting enzyme 2 (ACE2) in SARS coronavirus-induced lung injury. Nat Med. (2005) 11:875–9. 10.1038/nm1267 16007097 PMC7095783

[36] Meng J Xiao G Zhang J He X Ou M Bi J. Renin-angiotensin system inhibitors improve the clinical outcomes of COVID-19 patients with hypertension. Emerg Microbes Infect. (2020) 9:757–60. 10.1080/22221751.2020.1746200 32228222 PMC7170368

[37] Cai G Bossé Y Xiao F Kheradmand F Amos CI. Tobacco Smoking Increases the Lung Gene Expression of ACE2, the Receptor of SARS-CoV-2. Am J Respir Crit Care Med. (2020) 201:1557–9. 10.1164/rccm.202003-0693LE 32329629 PMC7301735

[38] Zhao Y Zhao Z Wang Y Zhou Y Ma Y Zuo W. Single-Cell RNA Expression Profiling of ACE2, the Receptor of SARS-CoV-2. Am J Respir Crit Care Med. (2020) 202:756–9. 10.1164/rccm.202001-0179LE 32663409 PMC7462411

[39] Ziegler CGK Samuel JA SK Nyquist IM Mbano VN Miao. SARS-CoV-2 receptor ACE2 is an interferon-stimulated gene in human airway. Cell. (2020) 181:1016–35.e19. 10.1016/j.cell.2020.04.035 32413319 PMC7252096

[40] Bunyavanich S Do A Vicencio A. Nasal gene expression of angiotensin-converting enzyme 2 in children and adults. JAMA. (2020) 323:2427–9. 10.1001/jama.2020.8707 32432657 PMC7240631

[41] Lambert DW Yarski M Warner FJ Thornhill P Parkin ET Smith AI. Tumor necrosis factor-α convertase (ADAM17) Mediates regulated ectodomain shedding of the severe-acute respiratory syndrome-coronavirus (SARS-CoV) receptor, angiotensin-converting enzyme-2 (ACE2). J Biol Chem. (2005) 280:30113–9. 10.1074/jbc.M505111200 15983030 PMC8062222

[42] Jia HP Look DC Tan P Shi L Hickey M Gakhar L. Ectodomain shedding of angiotensin converting enzyme 2 in human airway epithelia. Am J Physiol Lung Cell Mol Physiol. (2009) 297:L84–96. 10.1152/ajplung.00071.2009 19411314 PMC2711803

[43] Pedersen KB Chhabra KH Nguyen VK Xia H Lazartigues E. The transcription factor HNF1α induces expression of angiotensin-converting enzyme 2 (ACE2) in pancreatic islets from evolutionarily conserved promoter motifs. Biochim Biophys Acta. (2013) 1829:1225–35. 10.1016/j.bbagrm.2013.09.007 24100303 PMC3838857

[44] Reich HN Oudit GY Penninger JM Scholey JW Herzenberg AM. Decreased glomerular and tubular expression of ACE2 in patients with type 2 diabetes and kidney disease. Kidney Int. (2008) 74:1610–6. 10.1038/ki.2008.497 19034303

[45] Sama IE Ravera A Santema BT van Goor H Ter Maaten JM Cleland JGF. Circulating plasma concentrations of angiotensin-converting enzyme 2 in men and women with heart failure and effects of renin-angiotensin-aldosterone inhibitors. Eur Heart J. (2020) 41:1810–7. 10.1093/eurheartj/ehaa373 32388565 PMC7239195

[46] Li W Moore MJ Vasilieva N Sui J Wong SK Berne MA. Angiotensin-converting enzyme 2 is a functional receptor for the SARS coronavirus. Nature. (2003) 426:450–4. 10.1038/nature02145 14647384 PMC7095016

